# Antimicrobial resistance: a silent pandemic

**DOI:** 10.1038/s41467-024-50457-z

**Published:** 2024-07-23

**Keywords:** Antimicrobial resistance

## Abstract

Antimicrobial resistance (AMR) occurs when microorganisms (bacteria, viruses, fungi and parasites) no longer respond to antimicrobials, rendering these specific treatments ineffective. Subsequently, this narrows the options for clinical treatment and increases the risk of complications, hospital admissions, and mortality rates. Ultimately, infections become more difficult to treat. The concern of AMR is not new, yet the COVID-19 pandemic has further highlighted this global burden and raised questions regarding the preparedness for the fight against increasing cases of AMR. In a joint collaboration, *Nature Communications, Nature Microbiology*, *Nature Medicine, Communications Medicine* and *Scientific Reports* have launched a Collection and call for papers, inviting submissions of papers that advance our understanding of all aspects of AMR, as outlined in the Collection scope.

Antimicrobial resistance (AMR) is a global burden, indiscriminate of country border or income levels, and exacerbated by “lack of access to clean water, sanitation and hygiene, poor infection prevention and control in healthcare facilities, lack of awareness, and poor access to quality diagnostics, treatments and vaccines”^1^. AMR is estimated to have contributed to over 4.95 million deaths worldwide in 2019^2^, with an approximate economic burden of US$1 trillion in additional healthcare costs by 2050^3^.

Another pivotal concern of AMR is the impact it will have on human health and established modern medical procedures; the success of routine surgeries, organ transplantation and cancer chemotherapy relies on antibiotics that prevent bacterial infections. Without effective antimicrobial therapies reducing the risk of complications following these procedures, AMR jeopardises the advancements made in modern medicine, and we will see increased incidences of infection-relation complications and associated mortality risks.

The spread of AMR is considered a One-Health problem, given the unification of human and veterinary practices with environmental factors. At the human level, the misuse and overuse of antimicrobials, as well as inappropriate prescription in medicine, have contributed to the emergence and spread of AMR. The prophylactic use of antimicrobials in livestock agricultural settings and the resultant AMR microbes and resistance genes identifiable in animal excretions is one route in which AMR links to the environment; other routes include incorrect disposal of antimicrobials, contamination from wastewater, and spread from reservoirs and animal carriers within wildlife settings.

Tackling the problem of AMR requires efforts at various levels, from international to personal. International AMR surveillance is a valuable tool in analysing antimicrobial consumption and AMR spread and in informing related policies. Likewise, at a national level, it is important to ensure that antimicrobial usage within veterinary and farming sectors is appropriate. From a healthcare and pharmaceutical perspective, healthcare providers and physicians have a duty of care to provide appropriate antimicrobial prescription, and aseptic healthcare conditions, while at a personal level, patients have a responsibility to ensure compliance to prescribed antibiotic regimens. Pharmaceutical companies also have a duty to ensure replenishment of the antimicrobial pipeline, through offering of funding and incentives to researchers. Research efforts should not only be directed at the discovery and development of novel antimicrobials and vaccines, but also at improving sensitive and accurate diagnostic technology for the early prevention and treatment of infections, as well as continuing research into understanding the mechanisms of AMR development across pathogens.

With the launch of this Collection, we invite submissions of original research and commentary that advance our understanding of all aspects of AMR, from the surveillance of AMR in clinical and environmental settings to novel approaches in combatting or preventing AMR (i.e. antimicrobial or vaccine development). Authors wishing to submit to this Collection should select the option for the “Antimicrobial resistance: a silent pandemic” Collection on our submission system.
